# Patterns of Research Effort in Birds

**DOI:** 10.1371/journal.pone.0089955

**Published:** 2014-02-26

**Authors:** Simon Ducatez, Louis Lefebvre

**Affiliations:** Department of Biology, McGill University, Montréal, Québec, Canada; University of Lethbridge, Canada

## Abstract

Between species differences in research effort can lead to biases in our global view of evolution, ecology and conservation. The increase in meta-taxonomic comparative analyses on birds underlines the need to better address how research effort is distributed in this class. Methods have been developed to choose which species should be studied to obtain unbiased comparative data sets, but a precise and global knowledge of research effort is required to be able to properly apply them. We address this issue by providing a data set of research effort (number of papers from 1978 to 2008 in the Zoological Record database) estimates for the 10 064 species of birds. We then test whether research effort is associated with phylogeny, geography and eleven different life history and ecological traits. We show that phylogeny accounts for a large proportion of the variance, while geographic range and all the tested traits are also significant contributors to research effort variance. We identify avian taxa that are under- and overstudied and address the importance of research effort biases in evaluating vulnerability to extinction, with non-threatened species studied twice as much as threatened ones. Our research effort data set covering the entire class Aves provides a tool for researchers to incorporate this potential confounding variable in comparative analyses.

## Introduction

Birds and mammals are the most studied taxonomic groups in ecology and evolution [1]. This research bias, sometimes called “taxonomic chauvinism” [1], has been addressed in several studies [2]–[4]. Biases of this type can limit our understanding of global biodiversity and have negative consequences for conservation science [2]. On the other hand, extensive knowledge of whole taxonomic groups has the advantage of allowing large meta-taxonomic comparative analyses to test important questions in ecology, evolution and conservation over entire classes with high statistical power. Birds and mammals are indeed “model groups” for meta-taxonomic comparative analyses. In birds especially, the recent publication of complete phylogenies [5] now allows phylogenetically corrected analyses that include all 10 000 or so species.

**Figure 1:**
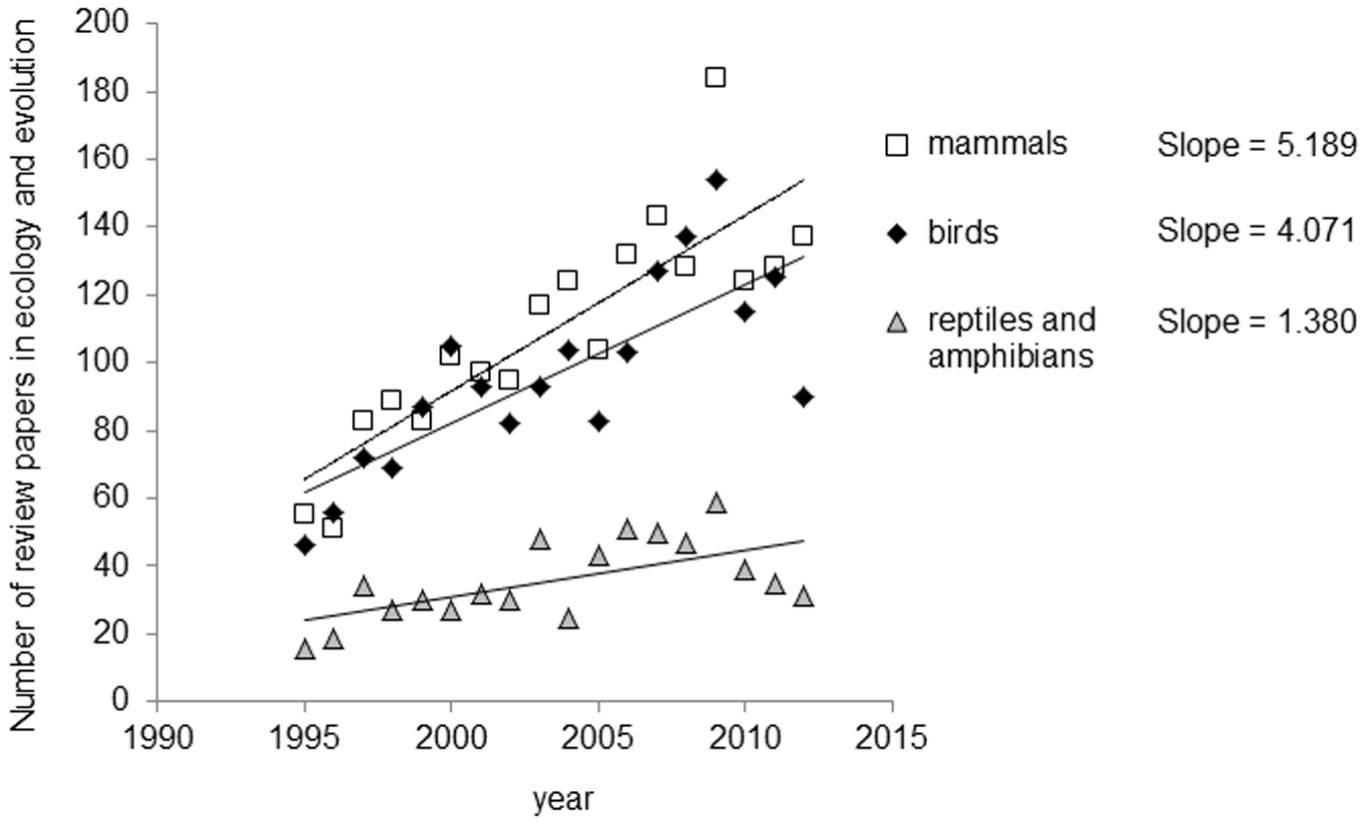
Number of reviews, comparative analyses and meta-analyses referenced on the Web of Science in ecology, evolution and conservation between 1995 and 2012 in birds, mammals and herptiles (amphibians and reptiles). The three slopes are significantly different from 0 (p<0.006 in the 3 cases), and the increase in the number of review papers across time is significantly smaller in amphibians and reptiles than it is in birds (p = 0.009) or mammals (p<0.001), but does not significantly differ between birds and mammals (p = 0.332).

In recent years, the number of reviews, meta-analyses and comparative analyses focusing on birds and mammals has been steadily increasing. A *Web of Science* search of the number of reviews, meta-analytic and comparative papers published on birds, mammals, or reptiles and amphibians in ecology, evolution, behavioral and conservation biology between 1995 and 2012 shows a strong increase in each of the classes, but one that is significantly stronger in birds and mammals than in reptiles and amphibians (Figure 1).

One key question in comparative research is the robustness of the data used in meta-taxonomic analyses. One determinant of this robustness is research effort: poorly studied taxa are more likely to have a larger sampling error in the estimate of their ecological and life history traits than heavily studied taxa. For example, the measurement of maximum longevity will be strongly affected by the low probability of encountering fewer and fewer long-lived individuals in a poorly studied species. This in turn will affect estimates of lifetime reproductive parameters, which have crucial roles in conservation or evolutionary biology. Research effort in bird conservation is already known not to reflect global geographic and taxonomic priorities [6], [7].

In this paper, we provide research effort data for all 10 064 species of birds currently listed in the IUCN database (see Table S1) and look at phylogenetic, ecological and life history variables that are associated with the trends in the whole class. Our long-term interest in research effort stems from the need to estimate biases of this type in our work on taxonomic differences in feeding innovations [8], [9]. Innovation rate is one of several measures based on counts of cognitive behaviors in the field, along with tool use, social learning and tactical deception [10]–[12]. For all these measures, assessment of cognitive behavior frequencies are likely to be affected by the probability of an observer witnessing them, which in turn depends in part on the number of studies conducted on this species, a variable that can be controlled (or analyzed, [13]) in comparative analyses [8], [10]–[12].

Estimates of many life history and ecological traits could similarly be biased by differences in research effort. Although methods have been developed [14] to choose which species should be studied to obtain unbiased comparative data sets, precise knowledge of research effort distribution is required to be able to properly apply them. Many factors are likely to bias research effort. Some species might be easier to study than others because, for instance, they are larger, have wider ranges, or occupy more accessible geographical areas. Others might show traits that are of greater interest to researchers, for instance, migration, large clutches or vulnerability to extinction. Here, we test whether research effort at the species level in the entire class Aves is associated with taxonomy (order, family, genus), phylogeny and eleven ecological and life history traits: biogeographic realm, insularity, habitat breadth, distribution range, population size, clutch size, generation length, longevity, body mass, breeding system and migratory behavior. Finally, we ask whether species that are vulnerable to extinction show research effort trends that differ from species that are not.

## Methods

### Research Effort Database

Global research effort was estimated for each of the 10 064 bird species listed on the IUCN website (including extinct species) using the Zoological Record database. This database covers over 5,000 serials, plus many other sources of information including books, reports, and meetings, and is thus one of the most exhaustive compilations of the zoological literature. We extracted, for each species, the number of publications referenced in this database between 1978 and 2008 (the extraction was made in June 2012). Our entire data set is presented as Table S1. We used the current Latin names included on the IUCN website as reference names for each species, and searches were made on keywords, abstracts and titles. To test whether this search method could in itself bias our data, we re-estimated research effort on 200 randomly chosen species using article titles only, and compared these estimates with the ones obtained with keywords, abstracts and titles.

As species and genus names are regularly modified with advances in molecular taxonomy, some species are known in the literature by different names, and their research effort is thus likely to be underestimated when only considering the currently used IUCN name. To test for this potential bias, we randomly drew 500 species in the whole class, and collected the different Latin names known for these species on the avibase.com website, an extensive information system containing bird name synonyms. We then re-assessed research effort for these species considering the different Latin names for each of them, and estimated the correlation between this new estimate of research effort and the one based on the current name only. Finally, we tested whether research effort could be biased by taxonomic stability itself, by asking whether the number of different names listed per species was associated with research effort.

### Taxonomic Biases

We first used Phylogenetic Linear Mixed Models (PLMM) with Markov chain Monte Carlo (MCMC) techniques using the R package MCMCglmm to estimate the proportion of variance in research effort (log-transformed) explained by phylogeny. The proportion of variance explained by phylogeny was calculated as the ratio VP/(VP+VR) with VP the variance explained by phylogeny and VR the residual variance. We used the phylogeny from [5] available on http://birdtree.org/. This website does not provide a unique consensus tree, but sample trees from a pseudo-posterior distribution. We randomly extracted 10 different trees, and ran one model per tree, providing 10 phylogenetic and residual variance estimates that we averaged to calculate the proportion of variance explained by phylogeny.

To identify the taxonomic level explaining the most important part of the variance in research effort, we then classified each species by its genus, family, and order, according to the classification used by the IUCN. We used Linear Mixed effects models to estimate the proportion of variance explained by each taxonomic level, using the lme procedure from the nlme R package. Research effort (log-transformed) was used as response variable, and order, family and genus were included as random effects, with genus nested in family, and family nested in order. We compared the AIC of models without any higher taxonomic levels, vs models including only order, vs models with family and order, vs models with genus, family and order. We estimated the proportion of variance explained by each taxonomic level calculating intra-class coefficients (ICC) for each of the 3 taxonomic levels, using variance estimates from the complete model (i.e. with the 3 taxonomic groups). Maximum likelihood was used to compare the AIC for different models, but we used Restricted Maximum Likelihood to get variance estimates used to calculate ICC, as advised in [15].

### Geographic Biases

We tested whether a species biogeographic realm predicted research effort by including biogeographic realm in a linear model with research effort (log-transformed) as the response variable. We used the Biogeographic realms classification from the IUCN, which identifies 13 realms. These data were not available for 4 extinct species, which were excluded from the analysis. We also tested whether insular and continental species had different research efforts, using island and continental status provided on the Birdlife website.

### Species Traits Biases

We tested whether 9 traits often studied in the literature could be associated with research effort. Extinct species (n = 134) and species classified as data deficient (n = 60) were excluded from these analyses. We first considered the association between research effort and habitat breadth, using habitat data from the IUCN. We expected species inhabiting a larger diversity of habitats to be more easily observable, and thus more often investigated. The IUCN provides a habitat classification scheme that defines 82 different habitat subtypes. Habitats were placed into 8 categories based on the categorization scheme of Bennett and Owens [16]: forest; woodland; scrub; tundra, moorland, and mountain; grassland, steppe, savannah, and agricultural; marine; marshland, freshwater habitats; and urban and suburban habitats, and we summed for each species the number of different categories it was recorded in (i.e. from 1 to 8) to obtain our measure of habitat breadth in 9870 species. We also considered distribution range (available for 9758 species; source = Birdlife website) and population size (available for 2945 species; source = Birdlife website) as we expected more widely distributed and abundant species to be more easily investigated. Body mass (available for 7703 species; source: [17] and Birdlife website) was also considered as larger species are more easily observable. Clutch size (available for 4954 species; source: [18]), breeding system (available for 9277 species; source: [19]) and migratory behavior (binary variable: migrant vs resident species; available for 9875 species; source: Birdlife website) were also considered as we expected species with larger clutches to be important targets in evolutionary studies (especially for studies in quantitative genetics and reproductive biology), species with particular breeding systems (e.g. brood parasites) to be more often investigated, and migrant species to have higher research effort as migration is a research area *per se*. Finally, as we expected research effort to affect longevity estimates, we considered longevity (available for 812 species; source: http://www.demogr.mpg.de/longevityrecords/0303.htm, see also Table S2) and generation length (available for 9147 species; source: Birdlife website) in our analyses. For continuous variables, we used Spearman’s rho to test for correlations between these traits and research effort, as some of the variables did not follow a normal distribution. A Wilcoxon test was used for the migratory behavior, and a chi-square test for breeding system.

We conducted a second set of analyses using Phylogenetic Linear Mixed Models (PLMM) with Markov chain Monte Carlo (MCMC) techniques using the R package MCMCglmm. These analyses allowed us to test whether phylogenetic biases and species traits biases were confounded, as trait values might not be randomly distributed according to phylogeny. Research effort (log-transformed) was included as response variable, and we included the trait of interest as fixed variable, building one model per trait. We included phylogeny as a random factor, using the phylogeny from [5] available on http://birdtree.org/. We randomly extracted 10 different trees, and ran one model per tree, providing p-values and effects estimates with standard errors showing errors due to phylogenetic uncertainty. Following Hadfield [20], we fixed the covariance structure and used poorly informative priors for the variances. For each model, the MCMC chains were run for 210 001 iterations with a burn-in interval of 10000 to ensure satisfactory convergence. A total of 1000 iterations were sampled to estimate parameters for each model. We checked that autocorrelation levels among samples were lower than 0.1.

### Extinction Risk

As research effort biases may have strong implications for conservation decisions, we also tested whether species extinction risk was associated with research effort. We used the IUCN Red List status as our measure of species extinction risk. We converted the risk categories into a binomial index with species classified under ‘Least Concern’ on one side, and threatened species on the other (CR, EN, NT and VU IUCN categories). We used a Wilcoxon test to compare threatened and ‘Least Concern’ species. Extinct (n = 134) and species with deficient data in the IUCN database (n = 60) were excluded from this analysis.

## Results

Research effort per species as estimated by the 1978 to 2008 editions of the Zoological Record varied from zero papers in 2217 species (for example the Tepui tinamou *Crypturellus ptaritepui*, the Choco trogon *Trogon comptus* or the white-capped fruit dove *Ptilinopus dupetithouarsii*) to more than 1500 papers each in species such as *Sturnus vulgaris, Anas platyrhynchos, Falco peregrinus, Parus major, Passer domesticus* and *Larus argentatus* (Table S1). In some cases (e.g. *Gallus gallus*, *Columba livia*, *Phasianus colchicus*, *Taeniopygia guttata*) studies in captivity inflate effort compared to species that are exclusively or most often studied in the field.

Estimating research effort from searches made with titles, keywords and abstracts, or exclusively with titles, gave similar estimates: the two search methods were highly correlated across our 200 randomly chosen species (Pearson correlation coefficient between the log-transformed research effort estimates = 0.899; p<0.001). Changes in taxonomic nomenclature also seem to have little effect on research effort estimates. Within our 500 randomly chosen species, research effort that includes multiple species names is very highly correlated with effort measured on the current name only (Pearson correlation on log-transformed research efforts = 0.958; p<0.001). In addition, the amount of taxonomic changes did not vary according to research effort, as illustrated by the absence of correlation between research effort and the number of different names per species (Spearman rho = −0.015; p = 0.738). Despite these points, we would still recommend that researchers interested in a particular set of species consider taxonomic revisions in addition to the current name provided in Table S1.

### Research Effort and Taxonomy

Phylogeny accounted for 74% of the variance (range = 73–75%) in research effort among species. Higher taxonomic levels explained a significant part of species variation in research effort. The model with the lowest AIC included all three taxonomic levels (AIC values for the different models: no taxonomic group included = 36304.05; including order = 34145.77; including family nested in order = 33498.06; including genus nested in family nested in order = 32587.38). Overall, the three higher taxonomic levels explained more than 50% of species variance in research effort, the effect of the order being the most important (34%; see Table 1). A species’ order, family and genus thus determine a large part of the variance in the research effort devoted to it.

**Table 1:** Intra-class Coefficients (ICC) for the different taxonomic levels and for the 3 levels together.

	ICC
Order	0.344
Family nested in order	0.067
Genus nested in family	0.135
Together	0.546

**Figure 2:**
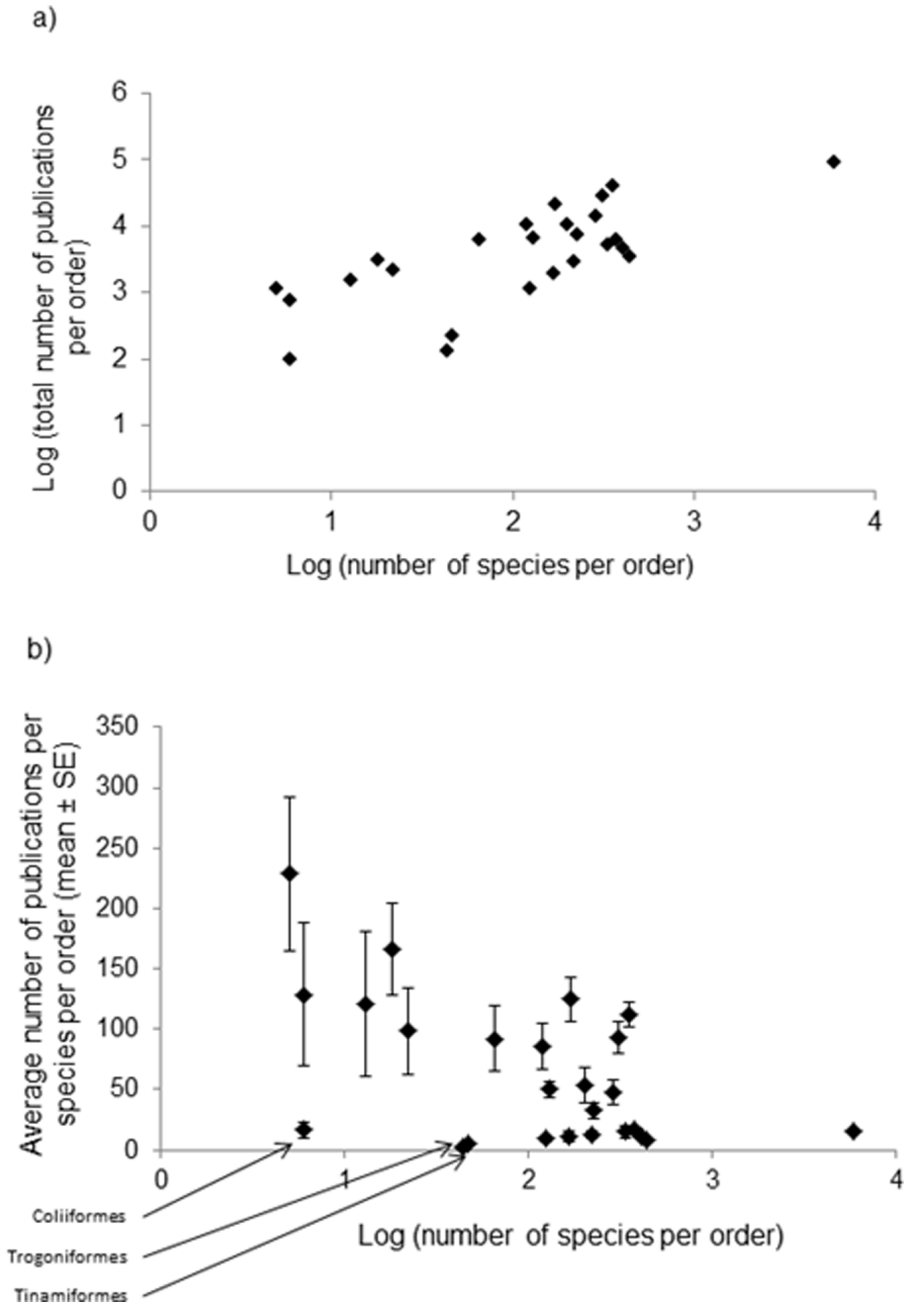
a) Relationship between the total number of publications per order and the number of species per order. b) Relationship between the average number of publications per species within each order, and the number of species per order. Orders with low species numbers and low publication number per species are identified for information (see Table 2 for details on each order).

As order explains the largest part of the variance in species level research effort, we investigated in more detail how research effort was distributed at that taxonomic level. The number of publications per order, number of species per order and average number of publications per species within each order are given in Table 2 (see also Figure S1). The total number of publications per order increased with order diversity (Spearman rho = 0.687; p<0.001), even if some orders with high species numbers were studied relatively rarely, such as Apodiformes, Piciformes, Psittaciformes and Columbiformes (see Figure 2a). At the level of species within orders, however, we found a negative relationship that reached borderline significance: species from smaller orders tended to have a higher mean research effort than species from larger orders (Spearman rho between the number of species per order and the average number of publications per species within each order = −0.354, p = 0.055, Figure 2b). Species from small orders such as the Gaviiformes (5 species) and the Sphenisciformes (18 species) had particularly high research effort (Table 2), while Trogoniformes, Tinamiformes and Coliiformes were understudied (see Figure 2b and Table 2).

**Table 2:** Research effort and species number in the different orders, ranked in alphabetic order.

Order	Publication number	Species number	Publication number per species (mean ± SE)
Anseriformes	21449	172	124.703±18.539
Apodiformes	3401	443	7.677±1.520
Caprimulgiformes	1140	125	9.120±2.335
Charadriiformes	39990	356	112.331±10.243
Ciconiiformes	10290	120	85.750±18.555
Coliiformes	99	6	16.500±6.350
Columbiformes	5090	336	15.149±4.940
Coraciiformes	2860	220	13.000±2.997
Cuculiformes	1872	167	11.210±3.779
Falconiformes	28831	312	92.407±13.107
Galliformes	13735	289	47.526±10.037
Gaviiformes	1142	5	228.400±63.834
Gruiformes	7331	226	32.438±6.726
Passeriformes	93614	5954	15.723±1.027
Pelecaniformes	6092	66	92.303±26.918
Phoenicopteriformes	773	6	128.833±59.165
Piciformes	4743	410	11.568±2.120
Pocicepediformes	2166	22	98.455±35.569
Procelariiformes	6543	131	49.947±6.787
Psittaciformes	6296	375	16.789±2.083
Sphenisciformes	2994	18	166.333±37.923
Strigiformes	10769	201	53.577±14.367
Struthioniformes	1567	13	120.538±59.927
Tinamiformes	215	47	4.574±1.216
Trogoniformes	133	44	3.023±0.756

### Geographic Biases

Biogeographic realm was significantly associated with research effort (*F*(12,9865) = 250.4; *p*<0.001), with European and North American birds studied much more often than species from any other realm (Table 3). Similarly, species inhabiting several realms were more studied than species endemic to a single realm (Table 3). In addition, insular species were much less studied than mainland species (mean publication number for insular species = 6.329±0.518; for mainland species = 33.315±1.403; Wilcoxon test: *p*<0.001). Using PLMM to take phylogeny into account did not affect this result.

**Table 3:** Publications per Biogeographic realm, ranked by decreasing number of publications per species.

Realm	Publication number	Species number	Publication number per species (mean ± SE)
Europe	2328	27	86.222±27.553
Several	213562	2673	79.896±3.749
North-America	14644	323	45.337±5.798
Australasia	8520	645	13.209±1.314
Africa	12535	1765	7.102±0.525
Eastern-Asia	12599	1917	6.572±1.066
Central-Asia	13	3	4.333±2.404
Caribbean	703	164	4.287±0.619
South-America	7706	2272	3.392±0.196
Oceania	295	144	2.049±0.283
Middle-East	47	23	2.043±0.424
Antarctica	5	3	1.667±1.202
Central-America	167	101	1.653±0.540

Four extinct species had an unknown biogeographic realm and are thus not included here. The low number of species in Europe, Central Asia, Middle-East, Antarctica and Central America is due to the fact that most species from these areas occupy several geographic areas, and were thus included in the category “several”.

### Biases Related to Species Traits

**Figure 3:**
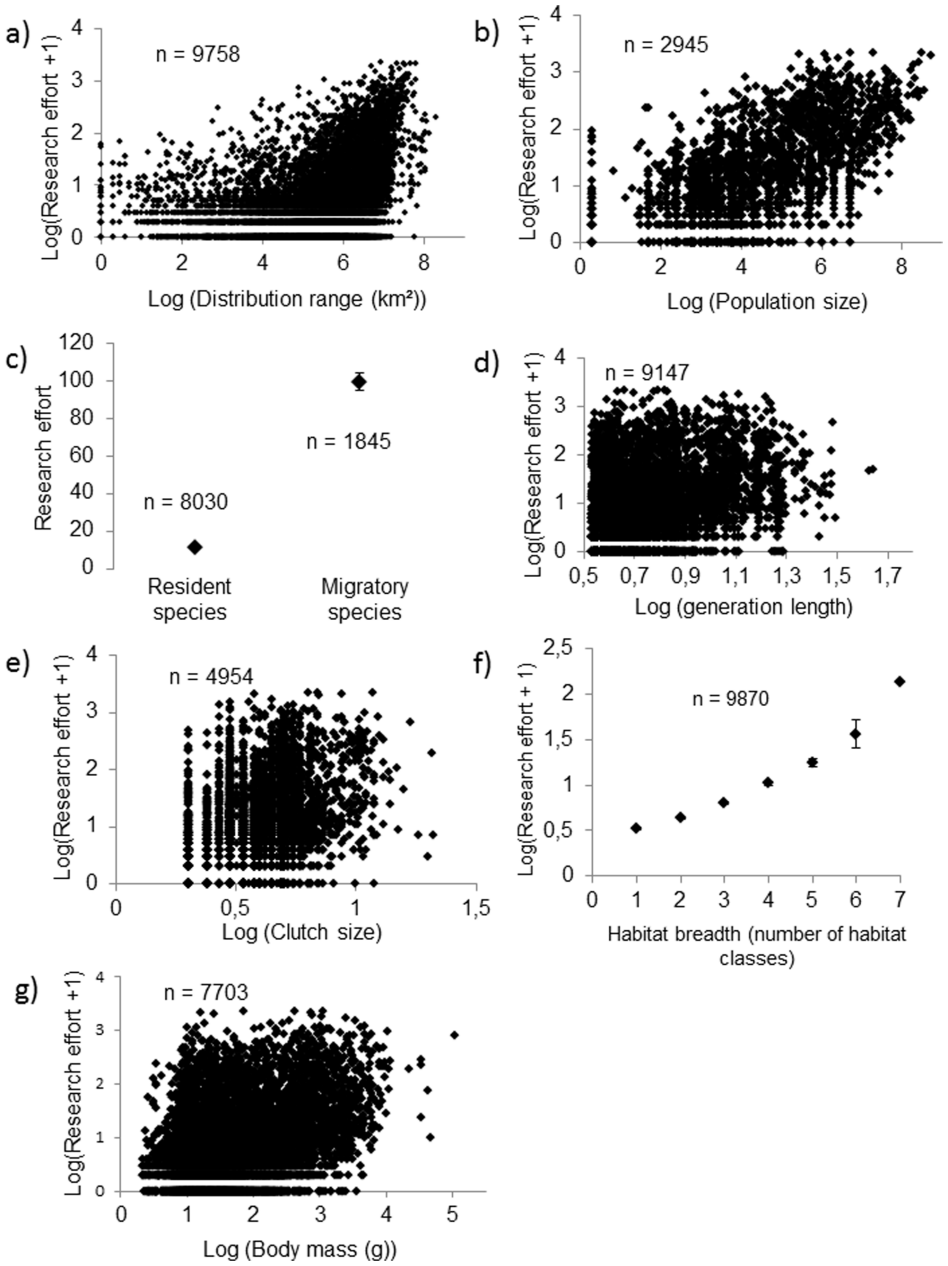
Relationship between research effort and species traits (panels c and f: mean ± se).

All the traits we tested were significantly associated with research effort. Research effort was significantly higher in species with a wider habitat breadth (Spearman rho = 0.276; p<0.001; n = 9878), species with a larger distribution range (Spearman rho = 0.441; p<0.001; n = 9758), species with a larger population size (rho = 0.538; p<0.001; n = 2945), species with larger clutches (Spearman rho = 0.337; p<0.001; n = 4961), species with a longer generation length (Spearman rho = 0.288; p<0.001; n = 9147), species with a greater longevity (rho = 0.327; p<0.001; n = 812) and species with a larger body mass (Spearman rho = 0.324; p<0.001; n = 7703; see Figure 3 and Table 4 for a summary of these results). Research effort also depended on breeding system (*F*(5,9319) = 3.043, p = 0.009): geothermal nesters and species with male-only parental care were less studied, species where parental care is provided by the two parents or where cooperation occurs had an intermediate research effort, whereas the most studied species were brood parasites and species without paternal care (see Table 5). Finally, migrant species had a higher research effort than resident species (mean research effort in migrant species = 98.790±4.656, n = 1845 species; mean research effort in resident species = 10.947±0.716, n = 8030 species; Wilcoxon test: p<0.001, Figure 3). Using PLMMs to take into account the effect of phylogeny on these relationships did not affect the results (results not shown).

**Table 4:** Correlations between research effort and species traits, ranked by decreasing correlation coefficient. n = species number.

Correlates of research effort	Spearman rho	*p*	n
Population size	0.538	<0.001	2945
Distribution range	0.441	<0.001	9758
Clutch size	0.337	<0.001	4954
Body mass	0.324	<0.001	7703
Generation length	0.288	<0.001	9147
Habitat breadth	0.276	<0.001	9870

**Table 5:** Differences in research efforts (mean publication number per species) according to species breeding system. n = species number.

Breeding system	Research effort (mean ± SE)	n
Female only	36.910±5.100	766
Brood parasite	29.878±12.587	90
Pair	28.831±1.266	7558
Cooperation	23.787±4.204	839
Male only	19.764±4.825	89
Geothermal	13.600±4.343	5

Variation between species in different life history traits is intercorrelated, so that one or a few traits might be the main drivers of all the correlations found between research effort and the tested variables. To control for this possibility, we included all of them in a single PLMM. Population size was strongly correlated with distribution range (Spearman rho = 0.723), and maximum lifespan with generation length (Spearman rho = 0.682). We thus excluded population size and maximum lifespan from this analysis to avoid colinearity problems, and because distribution range and generation length were known for a larger number of species than population size and maximum lifespan. Correlations between all other variables were lower than 0.3. All variables were standardized to a mean of 0 and variance of 1 so that their relative importance could be assessed on a common scale. Breeding system, as a categorical variable, could not be standardized, so we excluded it from the analysis. Note however that including it in the multiple regression model improved the DIC (ΔDIC = 3.55, with similar patterns of research effort differences among breeding systems as described above), but that including it or not did not affect the significance or the relative importance of the other fixed effects, except for body mass which became non-significant (p = 0.148). The final model, which is presented in Table 6, was run on the 4038 species for which we had data on all the traits included. The inclusion of phylogeny and biogeographic realm as random effects improved the model’s DIC (ΔDIC >294), although it did not affect the significance or the relative importance of the fixed effects. We thus only show the results for the model that includes phylogeny and biogeographic realm. All the variables included in the model significantly affected research effort (Table 6). Distribution range (pm = 0.424) and migratory behavior (pm = 0.414) had the strongest effects, whereas habitat breadth (pm = 0.152) and body mass had weaker effects (pm = 0.086). The effects of generation length (pm = 0.348) and clutch size (pm = 0.315) were intermediate.

**Table 6:** Phylogenetic Mixed Model explaining research effort as a function of 7 life history traits.

Variable	pm	CI	*p*MCMC
Distribution range	0.424	[0.385; 0.460]	<0.001
Migration	0.414	[0.371; 0.449]	<0.001
Generation length	0.348	[0.272; 0.406]	<0.001
Clutch size	0.315	[0.262; 0.369]	<0.001
Habitat breadth	0.152	[0.114; 0.188]	<0.001
Body mass	0.086	[0.035; 0.131]	0.003

Models were run with 10 different phylogenies, and posterior means (pm), CI (Confidence Interval) and p-values were averaged over the 10 models with 10 different phylogenetic trees (see text for details). n = species number. n = species number.

### Extinction Risk and Research Effort

Classifying species into threatened (CR, EN, NT and VU categories of the IUCN, n = 2387) or ‘Least Concern’ (LC, n = 7871), we found that research effort was twice as high for species of ‘Least Concern’ status as for threatened species (research effort for LC species = 31.362±1.385; for threatened species = 14.487±0.941; Wilcoxon test: p<0.001). Note that the inclusion of species classified as Vulnerable and Near Threatened in the “threatened” category is extremely conservative with regard to our prediction, and that including these species in the “unthreatened” categories yields similar conclusions (results not shown). The average research effort for CR, EN, NT and VU categories was similar (from 13.683 to 15.101). Using PGLMM to take into account phylogeny did not affect this result.

## Discussion

Taken together, our results show that research effort is not randomly distributed among the 10 000 plus species of birds and that all life history and ecological variables examined here show statistically robust (most with p<0.001) research effort differences. In addition, the phylogenetic position of a species and the order, family and genus in which it is classified predicts a large proportion of the variance in the research effort devoted to it, with little effect of recent changes in taxonomic nomenclature. These results are not surprising, as random variation in research effort would be difficult to achieve given the logistic constraints imposed by some habitats and geographic areas. Our study does not aim at correcting the methodological issues related to research effort trends, but rather at describing them and making the data available to other researchers.

All the life history and ecological traits we tested here were associated with research effort. It is worth noting that the relationship between research effort and many of our variables was continuous rather than stepwise (Figure 3). There is thus no obvious threshold effect in the data, where sharp changes in the number of studies would accompany specific or extreme levels (e.g. species that have worldwide distributions) of the variables studied here. Although species traits are inter-correlated, the associations between traits and research effort were not exclusively driven by one or a few specific traits, or by phylogeny or geography, as all the tested traits remained significantly associated with research effort when included in a multiple effects PLMM. We were however able to estimate their relative importance. Population size and distribution range were both strong predictors across the entire class Aves, a finding that is similar to the one obtained for European birds by [21] on the related topics of sample size and missing data. Both their results and ours likely arise from the fact that species with larger ranges and populations are easier to study. Ease of study might also explain why species with a wider habitat breadth have a higher research effort, and why insular species are less studied. The geographic variation in research effort is likely to be explained by the fact that research investment varies sharply across continents, with North America and Europe the most studied areas. This geographic bias is a subject of major preoccupation, as geographic areas containing a large part of areas of conservation priorities are under-studied (i.e. South and Central America, Oceania, Caribbean and Eastern Asia) compared to temperate areas [6], [7], [22]. This observation is confirmed by the finding that species classified as threatened by the IUCN were only half as studied as non-threatened species. Research effort is thus often a compromise between the relative importance of knowledge on different species and the difficulty of funding and carrying out studies on them. The geographic difference might also explain why migrant species had a research effort that was ten times higher than non-migratory ones, as a large proportion of migrant species occur in these overstudied temperate areas. The high research effort on migrant species is also likely to be explained by the fact that migration is in itself an important research topic. The relationships found between research effort and some life history traits might also be explained by ease of study [3]. Larger species are easier to observe. Similarly, studies in reproduction biology or quantitative genetics are easier to realize on species with large clutches.

Two of our results appear at first to be contradictory. First the proportion of variance in research effort explained by phylogeny (74%) and by higher taxonomic levels (order, family and genus, 54%) differs. This is likely due to the fact that when summing species level effort into orders, families and genera, the phylogenetic relatedness between species within a genus, genera within families and families within orders disappears. Phylogenetic distance between clades at each of these levels is taken as constant, removing a key component of variance. The second apparent contradiction is between total research effort per order and species number (positive relationship; Figure 2a) and mean effort per species and species number (negative relationship; Figure 2b). This is a consequence of the slope of the positive relationship in Figure 2a, where effort increases with order size, but at a lower rate in large orders compared to small ones.

It is important to point out that biologists would not necessarily benefit from sampling species randomly with respect to their evolutionary history. Because of particular life histories or ecological circumstances, some species are of special interest as research targets for biologists. Along with migrant species, brood parasites show a relatively high research effort, likely due to their original life history and the costs they may inflict on their host species. The finding that research effort is biased towards species from less diversified taxa might also be a function of the interest that researchers have in getting a more exhaustive view of evolutionary patterns. In contrast, we also identified groups of species with original life histories that had relatively low research effort. Among the different animal classes, for instance, a disproportionate amount of research on parental care is done on birds [4]. However, geothermal nesters and species with male only care only are relatively understudied, although their original breeding systems (which together features less than 100 species) make them interesting targets for evolutionary studies. Similarly, species from poorly diversified orders (thus likely to have an original evolutionary history) such as Coliiformes, Trogoniformes and Tinamiformes have a particularly low research effort, suggesting that they should be targets of major interest for future studies.

For some traits such as innovation rate [13] or longevity, differences in research effort might explain differences in trait estimates. In these cases, it is crucial to take research effort into account in comparative analyses. Whether a trait estimate is biased due to variation in research effort is not necessarily obvious, making it sometimes difficult to decide whether to correct or not the given trait by research effort. Habitat generalists are, for instance, expected to be more often investigated because they are easier to find in a variety of habitats. However, we might also expect more intensely studied species to be recorded in a larger variety of habitats, increasing their habitat breadth index, whereas poorly studied species would be attributed an artificially low habitat breadth index. Deciding whether to include or not research effort in comparative analyses is thus a difficult question, and one solution could be to consider both absolute trait values and trait values corrected by research effort in separate analyses.

In this paper, we provide a database of research effort estimates covering the entire class Aves. Our analyses show that research effort is not randomly distributed with respect to phylogeny, geography, extinction risk, ecological and life history traits in birds. An important follow up would be to test whether in threatened species, research effort is also biased towards species with specific traits, which could have strong implications for our general understanding of species responses to current global changes.

## Supporting Information

Figure S1 **Mean research effort per species within each order.**
(TIF)

Table S1 **Research effort database for 10 064 species.**
(XLSX)

Table S2 **Longevity dataset.**
(XLSX)
